# The use of intraluminal PRESERFLO stenting in avoiding early postoperative hypotony

**DOI:** 10.1007/s00417-024-06567-x

**Published:** 2024-07-06

**Authors:** Raoul Verma-Fuehring, Mohamad Dakroub, Ahmed Bamousa, Gunda Kann, Jost Hillenkamp, Daniel Kampik

**Affiliations:** Department of Ophthalmology, University Hospital Würzburg (UKW), Josef-Schneider-Straße 11, 97080 Würzburg, Germany

**Keywords:** Glaucoma, Filtering surgery, Preserflo MicroShunt, Intraluminal stenting, Hypotony

## Abstract

**Purpose:**

Postoperative hypotony following PRESERFLO MicroShunt (PMS) implantation is a frequent cause of complications such as choroidal detachment and hypotony maculopathy. This study aims at evaluating the impact of intraluminal stenting of the PMS during the early postoperative period.

**Methods:**

We retrospectively analyzed the data of 97 patients who underwent PMS implantation with intraoperative placement of a Nylon 10–0 suture as intraluminal stent (PStent) and compared the outcomes to those of an existing database of the traditional MicroShunt implantation technique (PTrad, *n* = 120). The primary outcome measure was the intraocular pressure (IOP) at one week postoperatively. As a secondary outcome measure, adverse hypotony, defined as an IOP ≤ 5 mmHg with significant choroidal effusion and/or anterior chamber shallowing or the presence of macular folds was also assessed. Additionally, the time to stent removal and the IOP one week after stent removal were reported.

**Results:**

Preoperative median IOP was 25.0 (20.5–30.3) mmHg in PStent and 25.0 (19.3–32.0) mmHg in PTrad (*p* = 0.62). One week after surgery, the median IOP dropped to 10.0 (8.0–13.0) mmHg in PStent and 7.0 (5.0–9.0) in PTrad (*p* < 0.01). At one month, the IOP was 12.0 (10.0–14.0) mmHg in PStent and 10.0 (8.0–11.0) mmHg in PTrad (*p* < 0.01). After 3 months, both groups showed similar median IOP levels of 11.0 (8.0–13.5) mmHg and 10.0 (9.75–13.0) mmHg in PStent and PTrad, respectively (*p* = 0.66). The presence of adverse hypotony was significantly lower in PStent compared to PTrad (6.2% vs 15.8%, *p* < 0.05). In PStent the stent was removed after 30.0 (21.0–42.5) days. One week after stent removal the mean IOP drop was 6.1 ± 0.5 mmHg (*p* < 0.01).

**Conclusion:**

In the early follow-up period, intraluminal stenting of the PMS appears to be safe and effective in controlling the IOP while reducing early postoperative hypotony. Surgical success is not compromised by stent placement. Based on our data, it is recommended to remove the suture two to six weeks after surgery for most patients with uncomplicated postoperative clinical findings.
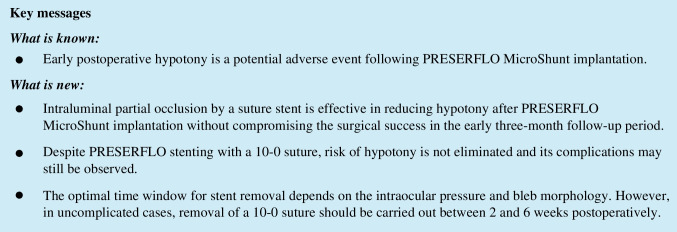

## Background

PRESERFLO® MicroShunt (PMS) implantation is a filtering procedure frequently carried out in the treatment of glaucoma. Although its intraocular pressure (IOP) lowering effect is slightly inferior to that of conventional trabeculectomy, it has a favorable safety profile [1]. It aims at avoiding postoperative ocular hypotony, which is a common potentially vision-threatening adverse event following glaucoma surgery. Several risk factors, such as male sex, older age, aqueous humor formation suppressing drugs, and an increased exposure to Mitomycin C (MMC) used during surgery have been correlated with an elevated susceptibility to this complication [2, 3].

Hypotony maculopathy and choroidal effusion (both hemorrhagic and serous) are serious complications of bulbar hypotony [4]. Hemorrhagic choroidal detachment is rare but associated with severe permanent visual loss. Serous choroidal effusion, on the other hand, is mostly self-limiting and possesses no substantial risk for permanent visual impairment. However, the latter can still cause temporary visual deterioration and is a risk factor for severe complications such as serous retinal detachment, appositional choroidals, or choroidal hemorrhage. Therefore, close monitoring is especially important in the early postoperative period [4]. Hypotony maculopathy with macular folds, on the other hand, is a complication that can permanently threaten visual acuity. It occurs due to distortion of the chorioretinal layers of the macula [5]. Studies have shown that the rate of this complication can be as high as 20% in patients who have undergone filtering glaucoma surgery [6].

The introduction of microshunts in filtering glaucoma surgery was promising in reducing the rate of ocular hypotony compared to traditional trabeculectomy. Microshunts have been shown to maintain an improved IOP control compared to trabeculectomy. However, the rate of hypotony still ranges from 13 to 29% [1, 7]. To further reduce the rates of hypotony and its complications, surgeons have historically been using non-resorbable sutures as intraluminal stents to prevent excessive outflow in the early postoperative phase [8, 9]. This approach is well practiced in glaucoma drainage devices such as the Molteno Implant, Baerveldt Glaucoma Implant, or Ahmed Glaucoma Implant [10–12]. Studies have shown that postoperative hypotony after PMS implantation can be successfully reduced by placing 8–0 and 10–0 sutures into the PMS lumen. The suture stents are then embedded in the corneal tissue for later removal at the slit lamp. Previous research has demonstrated the effectiveness of this technique in reducing postoperative hypotony to almost zero [8, 9]. Remarkably, no adverse events such as endophthalmitis or an increased rate of infections have been yet reported with regard to this technique. Although there is no official recommendation on stenting time, prior studies removed the intraluminal suture around two weeks postoperatively [8, 9].

In this study, we aimed to gather more precise data regarding the efficacy of PMS stenting. The outcomes of patients who underwent PMS stenting with intraluminal sutures were compared to those of patients who underwent the same procedure using the traditional technique (without intraluminal stenting). Furthermore, we aimed at assessing the optimal time window to stent removal.

## Methods

This study was approved by the institutional ethics committee at the University of Würzburg and adhered to the principles stated in the Declaration of Helsinki. We retrospectively analyzed the charts of 97 patients who underwent PRESERFLO® MicroShunt (PMS) implantation with intraluminal stenting at our institute between 05/2022 and 08/2023. Following a consensus among our glaucoma surgeons, all PMS implantations since 05/2022 have been performed using partial occlusion by an intraluminal stent. All patients were preoperatively informed about this off-label protective measure. Intraluminal stenting was carried out with the intraoperative placement of a Nylon 10–0 suture (Nylon Black Monofilament, Alcon Inc., Geneva, Switzerland) in a similar way as described by Lüke et al. [8]. The data from these patients were compared to those of an existing database of patients who underwent the traditional MicroShunt implantation technique (PTrad group, *n* = 120). These patients underwent MicroShunt implantation between 09/2020 and 05/2022. The indication for surgery was either an insufficiently controlled intraocular pressure (IOP) or intolerance to glaucoma drops. Only patients with primary open angle glaucoma (including normal tension glaucoma) and pseudoexfoliation glaucoma were included.

We defined the baseline IOP as the mean value of the IOP on the day of surgical indication and the IOP on the day of admission for surgery. All IOP values were obtained using Goldmann applanation tonometry. All patients underwent PMS implantation with application of intraoperative MMC 0.5 mg/ml (0.05%) for three minutes. Additionally, they received daily 5-fluorouracil injections of 10 mg/mL for the first five days after surgery. Prerequisites for the subconjunctival administration of 5-fluorouracil were a negative Seidel test and the absence of hypotony less than five mmHg, choroidal detachment, and corneal erosion. Preservative-free dexamethasone eye drops were prescribed for six months postoperatively, starting with six times daily and subsequently tapered by one drop per month. The early postoperative phase was defined as the first three months after surgery. Follow-up intervals were carried out week one, month one, month three postoperatively, as defined by the Guidelines on Design & Reporting Glaucoma Trials by the World Glaucoma Association [13], and one week after stent removal in accordance with the “preferred (follow-up) time window”.

The primary outcome measure was the IOP one week postoperatively, secondary outcome was the incidence of adverse hypotony. Adverse hypotony was defined as an IOP less or equal to five mmHg with severe choroidal effusion and/or anterior chamber shallowing or the presence of macular folds in OCT imaging. Choroidal effusion was classified as severe if bullae were present in two or more quadrants. High myopia, defined as myopia of ≤ -6.0 dpt or an axial length of ≥ 26.5 mm, was also obtained.

In the PStent group, the time to stent removal was also reported. The indication for stent removal was individually determined upon examination. Generally, a postoperative IOP of greater than 15 mmHg or the clinical onset of bleb scarring were considered indications for stent removal. All intraluminal stents were removed at the slit lamp after instilling one drop of topical anesthetic. These patients were examined again one week later.

Three months postoperatively, the number of pressure lowering compounds and need for revision surgery (bleb needling or PMS revision) were assessed. If either one was present, the intervention was classified as primary surgical failure.

SPSS (Version 29, IBM, New York, USA) was used for statistical analyses and G*Power (Version 3.1.9.6., Heinrich Heine University, Düsseldorf, Germany) for post hoc power analysis. Continuous variables were reported as mean ± standard deviation or median (interquartile range) as appropriate. The Kolmogorov–Smirnov test was used to test for normal distribution of continuous parameters. Means of normally distributed data sets were compared using t-tests, and those with non-normal distributions were compared using Mann–Whitney U tests. A chi-squared test was used to test for significant differences between dichotomous variables. Point-biserial correlation was deployed to test for correlations between nominal and metric variables. A p-value of less than 0.05 was considered statistically significant.

## Results

The statistical analysis was performed on 217 eyes (97 in PStent and 120 in PTrad). Baseline parameters did not differ significantly between both groups (Table 1). The mean age was 70.5 ± 10.7 years in PStent, and 71.1 ± 11.4 years in PTrad (*p* = 0.77). Both groups had similar baseline IOPs (PStent = 25.0 (20.5–30.3), PTrad = 25.0 (19.3–32.0), *p* = 0.62) and medication counts (3.0 (3.0–4.0) compounds and 4.0 (3.0–4.0) compounds in PStent and PTrad, respectively, *p* = 0.23).

**Table 1:** Baseline demographics of both groups. All parameters did not differ significantly. Data is shown as absolute values, median (interquartile range) or mean ± SD as appropriate

Parameter	PStent (*n* = 97)	PTrad (*n* = 120)	*p*-value
Age [y]	70.53 ± 10.7	71.1 ± 11.4	0.77
male/female [% / %]	53/44	42.5/57.5	0.08
Glaucoma Type [%] POAG PXG	69.1 30.9	78.0 22.0	0.16
High myopia [%]	18.6	16.7	0.72
Compounds	3.0 (3.0–4.0)	4.0 (3.0–4.0)	0.23
Baseline IOP [mmHg]	25.0 (20.5–30.3)	25.0 (19.3–32.0)	0.62

PStent = PRESERFLO® MicroShunt (PMS) implantation with intraluminal stent placement, PTrad = traditional PMS implantation, POAG = Primary open angle glaucoma, PXG = Pseudoexfoliation glaucoma, High myopia is defined as myopia of ≤ -6.0 dpt or an axial length of ≥ 26.5 mm, Compounds = pressure-lowering eye drops, IOP = Intraocular pressure in mmHg

**Fig. 1:**
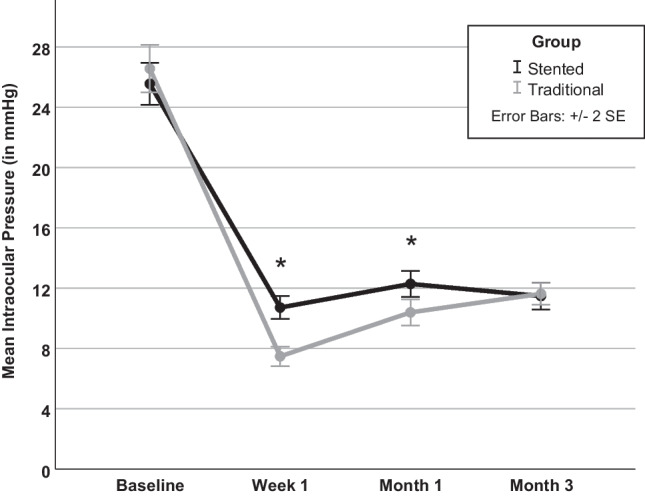
Course of the intraocular pressure (IOP) in the early postoperative period in both groups. Baseline IOP was similar in both groups (*p* < 0.01). Stent implantation led to improved IOP control in the first week postoperatively. At month three both groups showed similar IOP (*p* = 0.66). Stented = PRESERFLO® MicroShunt (PMS) implantation with intraluminal stent placement, Traditional = traditional PMS implantation, SE = standard error, * = values differ significantly.

**Fig. 2:**
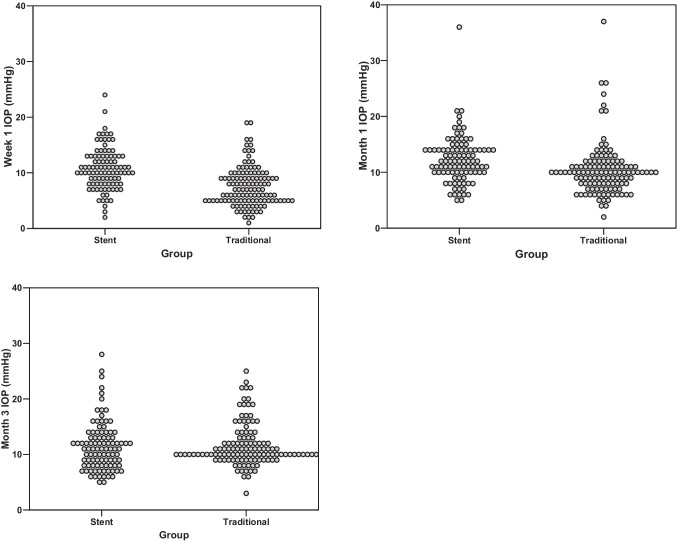
Intraocular pressure (IOP) in the early postoperative period (week 1, month 1, and month 3 postoperatively. Stent = PMS implantation with intraluminal stent placement, Traditional = traditional PMS implantation). At week one, we observed an upward shift of the IOP in the stented group compared to the traditional group. Even though PRESERFLO® MicroShunt (PMS) stenting reduced early postoperative hypotony, there were still a few cases of IOP values below 5 mmHg in the stented group

Figure 1 depicts the IOP values after surgery. One week postoperatively, the IOP dropped to 10.0 (8.0–13.0) mmHg in PStent and 7.0 (5.0–9.0) in PTrad (*p* < 0.01). At one month, these values increased to 12.0 (10.0–14.0) mmHg in PStent and 10.0 (8.0–11.0) mmHg in PTrad (*p* < 0.01). After 3 months, both groups showed similar IOP levels (11.0 (8.0–13.5) mmHg and 10.0 (9.75–13.0) in PStent and PTrad, respectively, *p* = 0.66). The postoperative outcomes are depicted in Table 2 and IOP outcomes in Fig. 2. The rate of postoperative serous choroidal detachment differed significantly in both groups (7.2% in PStent vs 19.2% in PTrad, *p* < 0.05). IOP readings of less than 6 mmHg were found in 7% of PStent patients and 35.8% of PTrad patients (*p* < 0.01). The occurrence of macular folds was similar in both groups (4.1% in PStent vs 5% in PTrad, *p* = 1.0). The incidence of adverse hypotony as defined above was significantly lower in PStent compared to PTrad (6.2% vs 15.8%, *p* < 0.05).

**Table 2:** Summary of postoperative results. Adverse hypotony was defined as presence of macular folds in OCT imaging or an IOP ≤ 5 mmHg with severe choroidal effusion and/or anterior chamber shallowing. Primary surgical failure was defined as the need for revision or the start of pressure lowering compounds. Data is shown as absolute values, median (interquartile range) or mean ± SD as appropriate

Parameter	PStent (*n* = 97)	PTrad (*n* = 120)	*p*-value
Week 1 IOP [mmHg]	10.0 (8.0–13.0)	7.0 (5.0–9.0)	< 0.01*
Month 1 IOP [mmHg]	12.0 (10.0–14.0)	10.0 (8.0–11.0)	< 0.01*
Month 3 IOP [mmHg]	11.0 (8.0–13.5)	10.0 (9.75–13.0)	0.66
Adverse hypotony [%]	6.2 (6/97)	15.8 (19/120)	< 0.05* (0.032)
Choroidal detachment [%]	7.2 (7/97)	19.2 (23/120)	< 0.05* (0.017)
IOP ≤ 5 mmHg at week 1 [%]	7.2 (7/97)	35.8 (43/120)	** < 0.01**
Macular folds [%]	4.1 (4/97)	5 (6/120)	1.0
AC shallowing [%}	2.1 (2/97)	3.3 (4/120)	0.7
Revision surgery [%]	6.3 (7/97)	18.0 (22/120)	< 0.05* (0.026)
Compounds at month 3	0.0 (0.0–0.0) 0.04 ± 0.3	0.0 (0.0–0.0) 0.2 ± 0.7	< 0.05* (0.005)
Primary surgical failure (revision or compounds) [%]	8.2 (8/97)	25.8 (31/120)	** < 0.01**

PStent = PRESERFLO® MicroShunt (PMS) implantation with intraluminal stent placement, PTrad = traditional PMS implantation, IOP = Intraocular pressure in mmHg, Compounds = pressure-lowering eye drops, AC = anterior chamber, Revision surgery = bleb needling or PMS revision, * = values differ significantly.

The PStent group had a higher surgical success compared to the PTrad group (*p* < 0.01). The need for revision surgery (bleb needling or PMS revision) was higher in PTrad (6.3% vs 18%, *p* < 0.05). After three months, the average medication count in the PStent group was lower than that of the PTrad group (median for both 0.0 (0.0–0.0), mean 0.04 ± 0.3 vs 0.2 ± 0.7, *p* < 0.05).

Our data did not show a significantly increased rate of hypotony in patients with high myopia in either group (*p* = 0.2 and 0.6 in PTrad and PStent, respectively). Also, no correlation was found between age and surgical failure (*p* = 0.3). A post hoc power analysis for IOP differences showed an achieved power of 99% and 81% at one week and one month postoperatively.

**Fig. 3:**
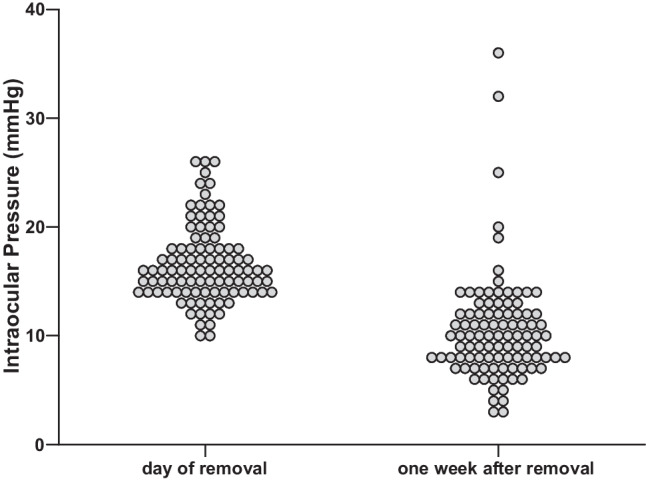
Scatter plot of the intraocular pressure (IOP) in the stented PRESERFLO® MicroShunt group (PStent) before and after stent removal. Removal resulted in a mean IOP drop of 6.1 ± 0.5 mmHg. The median IOP before and after removal was 16.0 mmHg (14.0–18.0) and 10.0 mmHg (8.0–12.0), respectively

In the PStent group the intraluminal stent was removed after 30.0 (21.0–42.5) days. Figure 3 depicts the IOP before and one week after stent removal. The median IOP before and after removal was 16.0 (14.0–18.0) and 10.0 (8.0–12.0), respectively. The minimum IOP recorded after stent removal was 3 mmHg and the maximum IOP 36 mmHg. One week after stent removal the mean IOP drop was 6.1 ± 0.5 mmHg (*p* < 0.01). After removal, 6.2% of patients developed adverse hypotony. Two patients who experienced adverse hypotony postoperatively also had adverse hypotony after stent removal. No cases of blebitis or endophthalmitis were reported. All intraluminal stents could be removed without complications at the slit lamp. In three patients the intraluminal stent was not removed until month three due to low intraocular pressure (< 12 mmHg in all three individuals) and therefore risk of developing hypotony after removal. Logistic regression showed no correlation between age or time of stent removal with the occurrence of adverse hypotony.

## Discussion

In this study we investigated the role of intraluminal stenting in patients who underwent PMS implantation at our department. Our data supports the hypothesis that PMS stenting with an intraluminal 10–0 suture is safe and effective in preventing adverse hypotony in the early postoperative follow-up period. In the first three months, patients who underwent PMS implantation with intraluminal stenting showed better early IOP control, less adverse hypotony, and no inferiority in terms of surgical success compared to the traditional PMS group. These results highlight the role of suture placement as a useful intraoperative adjunct in PMS implantation.

Compared to trabeculectomy, the PMS generally shows reduced complication rates and lower incidences of postoperative hypotony [1]. However, hypotony and its subsequent complications such as choroidal effusion and/or hypotony maculopathy are still observed with this technique [1, 7]. Although these events are often transient and self-limiting, they are still undesired. Vision threatening complications such as progressive choroidal effusion and macular folds should be taken seriously. Moreover, hypotony can impede or delay routine postoperative antifibrotic treatment with 5-fluorouracil, increasing the risk of surgical failure. Therefore, hypotensive patients often require closer observation and surgical intervention is sometimes necessary. Additionally, these events are also a burden for patients due to reduced visual acuity and frequent follow-up visits [5].

The studies by Lüke et al. and Lupardi et al. showed that the placement of an intraluminal suture as a flow restrictor helps stabilize postoperative IOP and minimize the rate of hypotony. In these studies, hypotony occurred in only one patient or not at all [8, 9]. Due to the increased rates of postoperative hypotony after PMS implantation, we have also started routinely stenting our PMS patients since 2023. In contrast to previous findings, only 6% of the stented PMS patients experienced adverse hypotony in our cohort. In two of our patients, we observed primary postoperative hypotony and the recurrence of hypotony after stent removal. The reason for this finding remains unclear. The extent of PMS occlusion may be a contributing factor. We deployed a 10–0 nylon suture (20–29 µm diameter) which occludes about 28–41% of a standard PMS (70 µm diameter). Lüke et al. utilized an 8–0 polyamide suture (diameter 40–49 µm) that occluded about 50–70% of the PMS lumen’s diameter [8]. Similarly to our study, Lupardi et al. stented the MicroShunt with a 10–0 nylon suture [9]. However, their population consisted of highly-myopic patients with a younger mean age of 62.95 ± 13.49 years. Hence, a faster rate of fibrosis and higher rate of aqueous humor production can be expected due to the younger age [14]. Our population was generally older and more heterogeneous resulting in presumably lower rates of fibrosis [15, 16]. This may be an additional reason for the observed difference in hypotony rates.

Another contributing factor may be the different dosing of Mitomycin C (MMC). It is well known that MMC has a toxic effect on the ciliary body and its usage may potentially decrease aqueous humor production [17]. Novel microshunts are mechanically designed to prevent IOP values below five mmHg assuming a physiological aqueous humor production rate of approximately 2.5 µL/min [18]. However, experimental data shows that the true resistance by the PMS itself would allow IOP below five mmHg [19, 20]. It is assumed that the sub-tenon space contributes to the outflow resistance, although the extent remains unidentified. It can be assumed that a decrease in aqueous humor production below 2.5 µL/min caused by iatrogenic factors, such as MMC, may disrupt the calculated flow-restriction and result in hypotony. It is possible that the relatively high concentration of MMC (0.05% for three minutes) that we deployed in our study, compared to other studies, influenced our incidence of postoperative hypotony. The Expert Consensus on the use of the PMS recommends the use of MMC with the concentration with which a surgeon has the best personal experience [21]. Schlenker et al. identified MMC concentrations 0.02% or lower as a potential risk factor for surgical failure after PMS implantation [22]. In the feasibility trial for the InnFocus MicroShunt (later termed PMS) 0.4% MMC was applied for three minutes with excellent rates of surgical success [18]. However, our PTrad group showed a hypotony rate similar to those that can be observed in different trials deploying lower MMC concentrations [1]. Hence, we do not assume that the application of MMC 0.05% increased the risk of hypotony. Additionally, another possible reason for early postoperative hypotony after PMS implantation may be aqueous humor leakage alongside the shunt due to a possible slight retraction of the PMS caused by an inappropriately sized scleral tunnel or pocket [20]. We did not actively observe any leakage in our PMS patients, however, it cannot be ruled out.

Compared to other studies, our time interval to intraluminal stent removal was longer at approximately 30 days postoperatively [8, 9]. So far, no consensus has been reached with regard to the optimal time window or target IOP for suture removal. In previous studies, this was carried out approximately two weeks postoperatively. As Lüke et al. occluded the PMS to a greater extent, an earlier rise in IOP with consecutive earlier need for stent removal was to be expected. The younger, highly myopic population of Lupardi et al. explains the earlier stent removal. Our population of stented PMS patients was not only larger but also more heterogeneous as it included non highly-myopic patients as well. Therefore, a average time window of approximately 30 days until stent removal may be more suitable for most glaucoma patients. Based on our data, which shows a hypotony rate of 6.2% after stent removal, it may be advisable to tolerate slightly higher intraocular pressure levels during the first 4–6 weeks after surgery. However, our data alone cannot answer this question, and the decision is likely to be an individual one. Interestingly, in all studies, including our own, an IOP drop of approximately 5–6 mmHg was observed after stent removal.

One limitation of this study is its retrospective nature. Additionally, the PMS implantations were performed by three different surgeons within our department. Moreover, our follow-up period was only three months. Long-term results on the impact of intraluminal 10–0 suture placement may reveal different outcomes with regard to surgical success. However, we were able to include a large number of patients in our statistical analysis because of our standardized follow-up protocol. Furthermore, this study was well-powered with respect to postoperative IOP control.

In conclusion, PMS stenting is efficient and safe in the early three-month follow-up period and can therefore be considered as an adjunct to the standard surgical procedure for PMS implantation. Nevertheless, despite stenting, we can still observe hypotony and its adverse effects after PMS stenting in our data. The ideal time frame or cut-off IOP for suture removal as well as the ideal intraluminal stent diameter remains a topic for future research. For the majority of PMS patients without severe complications, our data shows that it is advisable to remove the suture two to six weeks postoperatively. However, the timing of suture removal should be based on a multitude of factors such as presenting IOP, bleb morphology, and pressure fluctuations between follow-up visits.
